# Systematic review of evidence for the impact and effectiveness of the 1-3-7 strategy for malaria elimination

**DOI:** 10.1186/s12936-024-05200-w

**Published:** 2024-12-18

**Authors:** Nihal Sogandji, Anna Stevenson, Michael Y. Luo, Gao Qi, Richard J. Maude

**Affiliations:** 1 School of Clinical Medicine, University of Cambridge, Addenbrooke’s Hospital, Hills Rd, Cambridge, CB2 0SP UK; 2 Jiangsu Institute of Parasitic Diseases, Meiyuan Yangxiang 117, Wuxi, China; 3 Mahidol Oxford Tropical Medicine Research Unit, Faculty of Tropical Medicine, Mahidol University, Bangkok, 10400 Thailand; 4 Centre for Tropical Medicine and Global Health, Nuffield Dept of Medicine, University of Oxford, Oxford, OX3 7FZ UK; 5 The Open University, Milton Keynes, MK7 6AA UK; 6 School of Public Health, Li Ka Shing Faculty of Medicine, University of Hong Kong, Hong Kong, China

**Keywords:** Malaria, 1-3-7 strategy, Reactive case detection, Elimination

## Abstract

**Background:**

The 1-3-7 approach to eliminate malaria was first implemented in China in 2012. It has since been expanded to multiple countries, but no systematic review has examined the evidence for its use. A systematic review was conducted aiming to evaluate the impact and effectiveness of the strategy and identify key challenges and variations in its implementation across different countries.

**Methods:**

PUBMED, Cochrane Central Register of Controlled Trials (CENTRAL), MEDLINE, EMBASE, CABS Abstracts, LILACS, Global Health, Medrxiv, Biorxiv were searched for all studies containing 1-3-7 and articles included if they contained information on 1-3-7 impact, effectiveness, challenges and/or adaptations for implementation in different countries.

**Results:**

31 studies were included from China (19), Thailand (6), Myanmar (2), Tanzania (1), Cambodia (1), India (1) and Vietnam (1). During 1-3-7 implementation, malaria cases in China decreased by 99.1–99.9%, in Thailand by 66.9% during 2013–19, 65,1% in Cambodia during 2015–17 and 30.3% in India during 2015–16, with some differences in implementation. It was not possible to separate the impact of 1-3-7 from that due to other contemporaneous interventions. Implementing the 1-3-7 policy was largely effective, with reporting within 1 day in 99.8–100% of individuals in China and 36–100% in other countries, investigation within 3 days in 81.5–99.4% in China and 79.4–100% in other countries, and foci investigation within 7 days in 90.1–100% in China and 83.2–100% in other countries. Adaptations to 1-3-7 were described in 5 studies, mostly adjustment of the timing and/or definitions of each component. Key challenges identified included those related to staffing, equipment, process, and patient-provided information.

**Conclusion:**

Overall, the 1-3-7 approach was effectively implemented with a concomitant decrease in cases in malaria elimination settings, however, it was not possible to quantify impact as it was not implemented in isolation. Implementing adequate measures for testing, reporting, treatment, and containment is crucial for its success, which is dependent on the availability of resources, infrastructure, staffing, and consistent compliance across regions and throughout the year. However, achieving this nationally and maintaining compliance, especially at borders with malaria-affected countries, poses significant challenges.

**Supplementary Information:**

The online version contains supplementary material available at 10.1186/s12936-024-05200-w.

## Background

In 2022, it was estimated that the annual incidence of malaria was 249 million globally, up 5 million since 2021, as reported by the WHO World Malaria Report 2023. 95% of cases and 96% of the 608,000 deaths were located in the WHO African Region [1]. 80% of deaths in this region were in children under 5 years, with other high-risk populations being pregnant women and immunocompromised patients [1]. Since 2007, case numbers have decreased from an estimated 451 million [2]. In 2016, the WHO set a target of a 90% reduction of incidence and mortality by 2030 with the elimination of the disease in 35 countries. As evidenced by countries which have recently eliminated malaria, this can be achieved by a combination of high coverage for at-risk populations with effective preventative measures and improved detection and treatment of infected cases [1]. Prevention measures include insecticide-treated nets, indoor residual spraying and preventative treatment for infants and pregnant women [3]. However, vector control measures can be less effective outside of sub-Saharan Africa [4], and there is emerging antimalarial and insecticide resistance [5]. The WHO recommends that all suspected cases be confirmed with either microscopy or a rapid diagnostic test (RDT) [1]. This allows for case surveillance and reactive case detection (RACD), which has been utilised by at least 13 countries in the Asia Pacific region [6]. RACD involves contact tracing of close contacts of confirmed malaria cases and then testing and treating them with anti-malarials [7]. It has been widely suggested that RACD is necessary for malaria elimination. However, its success depends on the response time, resource availability and skills of the staff [7].

**Fig. 1:**
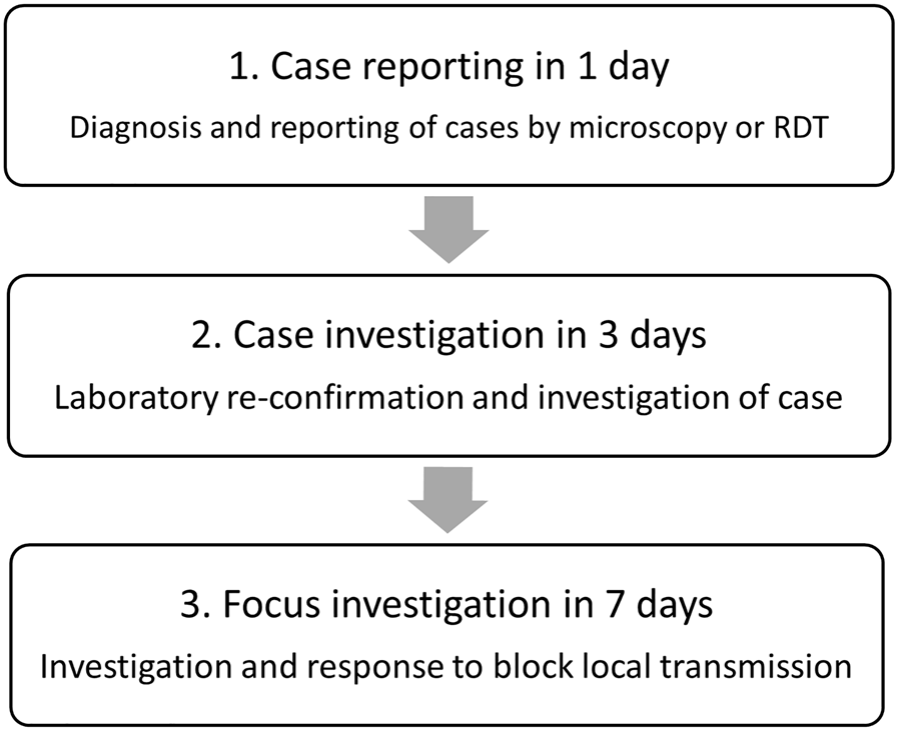
China’s 1-3-7 strategy

The 1-3-7 policy is a new malaria elimination strategy, which involves time targets for individual case-based interventions [6]. To successfully block local transmission, the 1-3-7 strategy focuses on 3 key points: case finding, looking for foci with local transmission and blocking local transmission before the next transmission occurs. It was first adopted in China in 2012 as part of the national malaria strategic plan to help them achieve their goal of eliminating malaria by 2020; subsequently, China was declared a malaria-free country by the WHO in 2021 after reporting zero indigenous malaria cases since 2017 [8]. The 1-3-7 policy has a series of targets which must be met. It requires the diagnosis and reporting of cases within 1 day (by microscopy or RDT); case investigation for all reported cases within 3 days (laboratory re-confirmation by microscopy or polymerase chain reaction (PCR) followed by epidemiological investigation for each laboratory re-confirmed case); and foci investigation and response to block local transmission within 7 days [9] (Fig. 1).

Since 2012, the 1-3-7 approach has been adopted by many other countries, including Thailand since 2016 [10], Myanmar since 2016 [11], Cambodia since 2015 [9] and Lao PDR since 2018 [12].

In spite of the successes seen in China, a previous scoping review by Yi et al. [13] identified potential issues and need for adaptation to implement the strategy in other countries. However, there has been no systematic review which examines the evidence for the true impact of the 1-3-7 elimination strategy on malaria burden and how its usage varies between different countries to evaluate suitability and identify potential areas for improvement in implementation. Given the interest of many countries in implementing the strategy to help achieve elimination, a systematic review was conducted aiming to identify the impact and effectiveness of the 1-3-7 strategy for malaria elimination, its successes and challenges, and the differences in its implementation between different countries.

## Methods

This systematic review was conducted in concordance with the Preferred Reporting Items for Systematic Review and Meta-Analyses (PRISMA) statement [14]. A completed PRISMA checklist is provided in Supplementary material 1. The protocol for this systematic review was registered on PROSPERO [15] (ID = CRD42023391742).

A systematic search for all studies related to the 1-3-7 strategy was conducted. Interventional and observational studies written in English were included whereas case reports and editorial pieces were excluded. The databases PUBMED, Cochrane Central Register of Controlled Trials (CENTRAL), MEDLINE, EMBASE, CABS Abstracts, LILACS, Global Health, Medrxiv, Biorxiv were searched for all studies containing 1-3-7 or reactive case detection in either their abstract or title until April 2024. The detailed search strategies can be found in Supplementary material 2.

The inclusion criteria were defined based on the PICOS framework [16], as follows:

1. **Participants/population:** Malaria endemic areas within China and other countries which have adopted or modified 1-3-7.
2. **Intervention(s), exposure(s):** 1-3-7 implementation or a 1-3-7 derivative in countries outside of China.
3. **Comparator(s)/control:** Either alternative malaria control strategies or pre-post studies for 1-3-7 introduction.
4. **Main outcome(s):** Incidence and/or prevalence of malaria, changes in transmissibility, changes of malaria infections in the population, compliance with program when considering 1-3-7 derivatives and changes from the standard 1-3-7 template.
5. **Study characteristics:** Interventional studies (e.g. randomized, quasi-randomized, pre/post studies), observational studies (e.g. cohort, case–control) and qualitative studies. Exclusion criteria were studies not in English, case reports and commentaries.

After this primary search, screening of abstracts was conducted using Rayyan [17], a systematic literature review software, independently by two researchers (NS and AS) who checked if the abstract met the inclusion and exclusion criteria for the three main questions. I.e. whether they were focused on 1-3-7 impact, effectiveness, and/or adaptation for implementation in different countries. If there was disagreement, a third independent reviewer (ML) made a final decision. After the abstract screening, this process was repeated with the full texts of the remaining articles.

References were snowballed to reduce the risk of missing important references that were not found in the searches. The standard inclusion and exclusion criteria were applied to each reference from all the included papers. This was also done by two researchers (NS and AS) independently.

Papers included following screening were assessed for risk-of-bias using the ROBINS-I [18] tool by a single researcher (ML) to assess the current state of research in the field and the quality of the evidence that can be drawn from them. No controlled studies were identified for inclusion in this review.

Following this, study characteristics including country of origin, quantitative/qualitative research, adaptations to 1-3-7 strategy and outcome data reported were extracted. Numerical data was extracted to develop a quantitative description of the impact and effectiveness of 1-3-7. Due to the heterogeneity in methods of data collection and types of data recorded, a meta-analysis was not performed. Instead, each study’s reported outcomes were tabulated against outcomes of interest for data synthesis and a narrative summary of the results provided in the manuscript. Furthermore, a qualitative analysis of the papers was performed describing how 1-3-7 was implemented, what were the challenges faced, and how 1-3-7 was adapted for implementation in different countries.

## Results

**Fig. 2:**
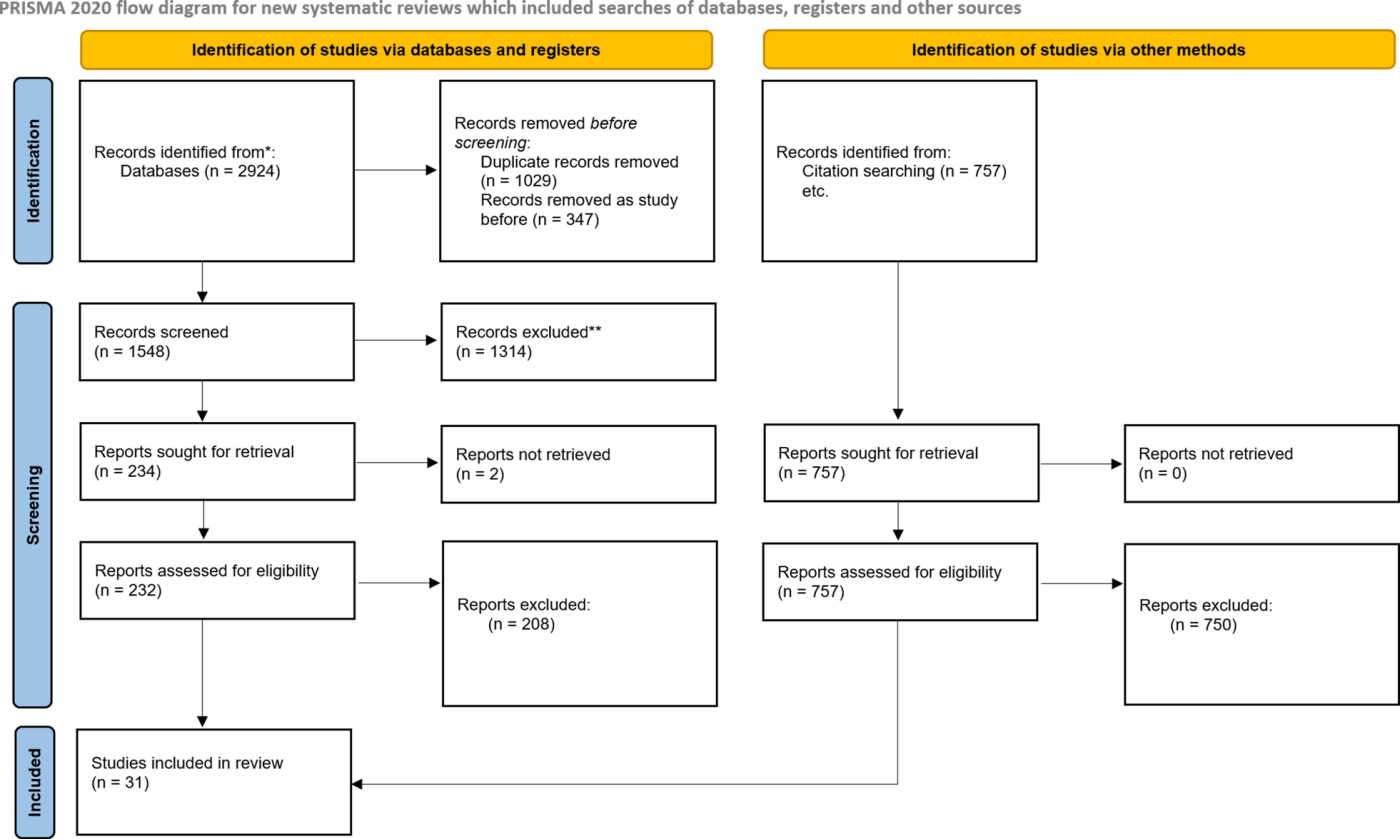
PRISMA flow diagram screening of studies [14]

Screening of papers from databases and snowballing was completed on 23rd of April 2024. Following screening, 2 papers could not be retrieved and 31 studies were included in the study (Fig. 2). The characteristics of each study can be found in Supplementary material 3 and extracted quantitative data in Supplementary material 4.

In addition, screening revealed Mlacha et al., which assessed the impact of the 1,7-malaria Reactive Community-based Testing and Response (1,7-mRCTR) which was implemented in the Rufiji District of Southeastern Tanzania from September 2015 to June 2018 [19]. This strategy involved reporting of confirmed case within 1 day and carrying out focal treatment in malaria-endemic villages within 7 days. However, this paper was excluded as this was not a modified 1-3-7 strategy aiming for malaria elimination, but a strategy inspired by 1-3-7 with the objective of reducing malaria burden in this high transmission area by 30% [19].

19 studies were conducted in China, 6 in Thailand, 2 in Myanmar, 1 in Cambodia, 1 in India, 1 in Vietnam and 1 in Tanzania (Table 1).

**Table 1:** Characteristics of included papers

Characteristic		Number of reports	References
Country	China	19	[6, 20–22, 25–28, 29, 30, 35–38, 40, 44–46]
	Thailand	6	[10, 23, 31, 39, 47, 48]
	Myanmar	2	[11, 32]
	Cambodia	1	[9]
	India	1	[24]
	Vietnam	1	[33]
	Tanzania	1	[34]
Study type	Qualitative	4	[11, 38, 39, 41]
	Quantitative	18	[6, 10, 20–25, 30, 31, 34–37, 40, 46, 47, 48]
	Both	9	[9, 26, 27, 28, 29, 32, 33, 44, 46]
Intervention	1–3-7	26	[6, 9, 10, 20–25,25–31, 35–41, 44–48]
	Adaptation	5	[10, 24, 32, 33, 34]
Measure of Impact	# cases	14	[6, 9, 20, 22, 24, 25, 26, 30, 35, 40, 45, 46]
	Rc	1	[22]
	# active foci	1	[23]
	Annual parasite incidence (API)	1	[9]
	None	16	[10, 11, 27, 28, 29, 31–34, 36, 38, 39, 41, 44, 47, 48]
Measure of effectiveness	% of cases following different steps of strategy timeline	16	[6, 10, 22, 23–34]
	Knowledge of 1–3-7	1	[41]
	None	14	[9, 11, 20, , 35–41, 45–48]

*Rc* case reproduction number

The detailed risk of bias assessment results are provided in Supplementary material 5. Using the ROBINS-I tool, 1 study was found to have serious risk of confounding bias. All the other studies were found to carry low risk of bias in all domains except for the domain of deviation from protocol, where all the studies were found to have a moderate risk of bias.

### Impact

#### China

The papers included in this study concluded that China’s elimination efforts have been successful, with a sharp decline in indigenous malaria cases of 99.1% (2010, n = 4262; 2015, n = 40) as reported by a retrospective analysis of active foci from 2010 to 2015 [20], and a decline of 99.9% reported from 2010 to 2017 by another retrospective evaluation [21]. Another, looking specifically at cases reported in Yunnan province during 2011–2016, due to high transmission across its borders and strong agricultural focus, found that the case reproduction number (Rc) for local cases caused by each of the *Plasmodium* species had decreased, with no Rc > 1 reported from 2014 onwards [22]. This decline was greater in the central parts of the province, with more cases reported in the regions bordering malaria-endemic countries. Finally, in 2017, zero indigenous cases were reported, with a total of only 2675 malaria cases, of whom 99.9% were imported, with the remaining cases infected via blood transfusion in the provinces of Jiangsu (n = 2) and Guangdong (n = 1) [21]. However, it was not possible to quantify impact from only 1-3-7 in these studies as the 1-3-7 strategy was strictly implemented simultaneously in all malaria endemic areas, thus there was no counterfactual for comparison.

#### Thailand

As part of Thailand’s National Malaria Elimination Strategy (2017–26), the 1-3-7 approach was introduced in 2016, defined as case detection in 1 day, case investigation in 3 days, and foci investigation in 7 days and aiming for zero indigenous cases by 2024 [10]. There was a decline in the number of indigenous cases by 78.4% from 17,553 to 3,787 and a decrease in the number of active foci from 2,227 to 700 [23]. However, there has been variable success among different foci which has been largely attributed to the disparities in the level of participation in the 1-3-7 programme as well as the higher numbers of imported cases in regions bordering malaria-endemic countries [10, 23].

#### Myanmar

There was no evidence of impact of 1-3-7 provided in the papers from Myanmar.

#### Cambodia

The 1-3-7 strategy was implemented in Sampov Loun, Cambodia, in 2015 with the aim of elimination by 2025. 1-3-7 was defined as case reporting within 1 day, case investigation within 3 days and foci investigation and response within 7 days, even though in practice case investigation and response were usually done simultaneously to give a modified strategy of 1-2-2 or 1-3-3 [9]. There was a sharp decline in the number of cases reported in the region by 34.9% from 519 in 2015 to 181 in 2017, and the annual parasite incidence in the region fell from 3.21 per 1000 population to 1.06 per 1000 population during the same period [9]. No indigenous cases have been reported since March 2016 in Sampov Loun [9]. The strategy was then expanded to the provinces of Battambang, Maung Russei, Thmar Koul and Pailin [9].

#### India

A modified 1-3-7-14 strategy in Mangaluru city, Karnataka, India coincided with a reduction of malaria incidence by 30.3% within 2 years [24]. The strategy added follow-up after 14 days to check for completion of radical treatment with primaquine for *P. vivax* cases.

#### Vietnam

There was no evidence of impact of 1-3-7 provided in the paper from Vietnam.

#### Tanzania

There was no evidence of impact of 1-3-7 provided in the paper from Tanzania.

### Effectiveness

Table 2 summarises the effectiveness for 1-3-7 implementation for each country.

**Table 2:** Percentages of cases reported in day 1 and investigated by day 3 and foci investigated by day 7 before and after implementation of the 1-3-7 strategy

Country	Citation	Case reporting		Case investigation		Focus investigation	
		Within 1 day		Within 3 days		Within 7 days	
		Before strategy	After strategy	Before strategy	After strategy	Before strategy	After strategy
China	Zhou et al. 2015 [6]	–	100%	–	97%	–	96.30%
	Routledge et al. 2020 [22]	–	–	–	–	–	
							96%
	Wang et al. 2017 [29]	–	–	–	81.50%	–	–
	Huang et al. 2021 [25]	–	100%	–	95.60%	–	97.90%
	Cao et al. 2022 [26]	–	100%	–	99.40%	–	98.30%
	Li et al. 2021 [27]	–	99.80%	–	–	–	94%
	Feng et al. 2014 [28]	–	100%	–	98.05%	–	–
	Feng et al. 2016 [30]	–	–	–	92.40% (2013)	–	–
					99.70% (2014)		
Thailand	Sudathip et al. 2021 [10]	18.20%	80.70%	73.80%	97.80%	56.50%	83.20%
	Sudathip et al. 2020 [23]	–	80%	–	80%		80%
	Lertpiriyasuwat et al. 2021 [31]	24.40%	87.80%	58.00%	94.70%	37.90%	84.10%
Myanmar	Kyaw et al. 2018 [32]	–	–	–	95.50%	–	96.60%
Cambodia	Khean et al. 2020 [9]	50%	100%	20%	100%	35%	~ 100%
India	Baliga et al. 2019 [24]	–	36%	–	80%	–	–
Vietnam	Oo et al. 2023 [33]	–	83.7%	–	79.4%	–	–
Tanzania	Mkali et al. 2023 [34]	36.1%	72.7%	79.5%	91.8%	59.8%	92.4%

#### China

In China, after implementation of 1-3-7, diagnosis and reporting of cases was completed in 1 day in 99.8–100% [6, 25–28] of cases reported, case investigation was completed in 3 days for 81.5–99.7% [6, 25, 26, 28–30] of cases, and foci investigation was completed in 7 days for 94.0−98.30% [6, 22, 25–27] of all cases reported. No data were reported on effectiveness of these components before implementation of the 1-3-7 strategy.

#### Thailand

In Thailand, all steps of the strategy were implemented within the recommended timeframes in over 80% of the cases, which was much higher that the equivalent figures prior to the implementation of the 1-3-7 strategy [10, 23, 31].

#### Myanmar

The National Malaria Elimination Programme (NMEP) introduced the 1-3-7 strategy in Myanmar in 2016, with the aim of indigenous *Plasmodium falciparum* malaria elimination by 2030. It was initially introduced in 6 low-endemic states or regions with the aim of rolling it out to the remaining 9 by the year 2030 [11]. The 1-3-7 strategy was similarly defined as notification of each case within 24 h after confirmation by Village Health Volunteers (VHV) and reporting over the phone, case investigation within 3 days and response and control within 7 days [32].

Unlike in China, the most problematic aspect of the implementation strategy was case detection within 24 h, where data on time and day of reporting was not kept, so the percentages of cases notified within 24 h could not be calculated [32].

Case investigation was completed within 3 days for 95.5% of cases and foci investigation and response was completed within 7 days for 96.6% of cases [32].

#### Cambodia

All aspects of the strategy carried out in Sampov Loun met the targeted timelines for nearly 100% of cases [9].

#### India

The modified 1-3-7-14 strategy in Mangaluru city, Karnataka, India, was implemented as part of a field trial during 2014–17 [24]. This was supported by digitalisation of surveillance data and introduction of a Geographic Information System.

36% of cases were reported within 1 day with 24% of cases reported later than 3 days. The proportion of cases investigated within 3 days was 80%. No data were reported on effectiveness at 7 or 14 days. Issues with the internet service were reported to be responsible, especially at smaller health facilities that may also be suffering from staff shortages.

#### Vietnam

As per the Reactive Surveillance and Response (RASR) Strategy, in Vietnam case reporting and investigation where supposed to be completed in 2 days and foci investigation and response in 7 days. In 2020, case reporting with a paper-based system was completed in 2 days for 63.8% of cases with no information provided on case investigation timelines. In 2021, following the introduction of an electronic reporting system, case reporting within 2 days rose to 83.7% and case investigation was completed in 2 days in 79.4% of cases [33].

#### Tanzania

In the Zanzibar region of Tanzania, a low transmission setting, 1-3-7 was first implemented in 2012, following which the percentage of cases reported within 24 h rose from 36.1 to 72.7% by 2021, the percentage of households visited within 3 days rose from 79.5 to 91.8%, and the percentage of foci investigated within 7 days rose from 59.8 to 92.4% [34].

### Challenges

A range of challenges was described in the included papers regarding the implementation of the 1-3-7 strategy.

A significant challenge to malaria elimination is importation of cases from bordering endemic countries. For example, while there was a significant reduction in the total malaria cases in China since the launch of the national action plan for malaria elimination (2010-2020) in 2010, there has been a significant increase in the proportion of imported cases [28], with the success in reducing malaria cases not being seen to the same extent in provinces which neighbour malaria-endemic countries [27, 35–37]. After the introduction and national roll-out of the 1-3-7 strategy in China in 2012, cases became concentrated along the Yunnan-Myanmar border [27]. Multiple explanations have been proposed for this. One is the very high number of people crossing the border each day for reasons ranging from healthcare to education to employment; indeed, malaria prevalence in Myanmar was strongly correlated with the total number of cases in Yunnan province [27]. Yunnan province has a history of elevated malaria case numbers and has faced a series of challenges to 1-3-7 implementation. These included higher rates of poverty, leading to lower use of insecticides and nets, inaccessible village infrastructure, inappropriate health-seeking behaviour of the inhabitants with higher illiteracy rates and the presence of migrant workers who travel back and forth between Yunnan and malaria-endemic countries [30].

The latter was also a common issue in other provinces, for example Shanxi which has reported longer delays between fever onset and presenting at healthcare services than the average for China, with imported cases from Africa and Southeast Asia among migrant manual labour workers [36]. Furthermore, these workers frequently relied only on the care they received from the healthcare authorities of the endemic country they imported the infection from and did not inform the services here in Shanxi, where county-level care suffers from lack of experience dealing with malaria cases [36]. Similarly, a dramatic decline in the number of indigenous cases was reported, along with a steep increase in imported cases in Shandong province [37].

Similar problems were seen in Cambodia, where foci investigation suggested that focusing reactive case detection on travellers and mobile and migrant populations may be a more efficient use of resources for the future [9].

#### Challenges in case reporting within 1 day

That China has been able to complete case confirmation in 1 day in nearly all malaria cases since 2010 has, in part, been possible due to malaria being a notifiable disease, with healthcare professionals responsible for recording cases on the China Information System for Disease Control and Prevention within 24 h [29]. So far, China has had tremendous success in case reporting, though how this will change with decreased focus on malaria and consequently the vigilance of healthcare workers and the challenge of imported cases remains a concern. For example, healthcare staff’s knowledge about 1-3-7, as reported by a survey conducted in Jiangsu, was only 63.2% in 2016 [38].

In Thailand, all steps of the strategy suffered from logistical challenges including poor phone and network signal and difficulty of transport to rural areas especially during the rainy season [39].

In Myanmar, case reporting within 1 day was the most problematic aspect of the strategy [11]. This was largely due to the absence of a central, real-time reporting system, instead relying on reporting of malaria cases over the phone, the vast majority of whom are diagnosed at primary health centres in Myanmar. This was exacerbated by the poor mobile phone signal in certain remote areas. Furthermore, delays in migrant populations seeking healthcare also delayed case detection, similar to in China. Finally, the lack of a legal requirement for reporting these cases within 1 day by healthcare staff, unlike in China, may have also contributed to the delays in this step.

In Vietnam, an important barrier to timely case reporting was the limited mobile phone network coverage in forests and field sites outside the villages, meaning healthcare staff could only report these cases once they returned back to the villages [33].

In Zanzibar, Tanzania, challenges in case reporting included difficulties in securing hardware replacements for their online reporting system, difficulties with software viruses and long-term maintenance and limited staff availability especially during peak malaria transmission periods [34].

#### Challenges in case investigation within 3 days

Investigation of cases was impacted by unavailability of trained healthcare staff and appropriate equipment, e.g. for microscopy, PCR, etc. In China, while RDTs and microscopy were used to detect and classify species at local level, more advanced testing methods requiring higher skill, e.g. PCR, were used in provinces for cases where parasites were not detected in blood, when identification of species was uncertain by microscopy [40]. In these cases, loss of data or delays during transport to provinces [21] were identified as problems. Classification of cases suffered from further difficulties; for example, determining whether a case is indigenous or imported relies on patient reporting, whereas patient history may not be obtained in sufficient detail and may be subjective or unreliable. Likewise, classifying cases as reinfection or relapse proved to be especially difficult for infections with *P. vivax*, as with this species, relapse of infection can occur long after the patient has left the endemic region where the infection was contracted [41].

In Myanmar, case investigation suffered from delays stemming from inadequate transport links, limited knowledge of healthcare staff regarding malaria case classification as well as unavailability of staff in certain regions [11].

In Cambodia, challenges associated with case investigation included maintaining healthcare staff’s willingness to participate in the strategy, especially during holiday periods, and difficulties dealing with imported cases [9].

In Vietnam, according to staff survey responses, one of the barriers to timely case investigation was difficulty in contacting the identified cases and the time required to travel to remote areas [33].

#### Challenges in foci investigation within 7 days

In China, an important part of the focus investigation and response is determining the risk of onward transmission [41]. According to China’s national malaria elimination strategy, tackling type 1 foci, characterised by local transmission, requires mass drug administration within the focus and indoor residual spraying. In type 2 foci, with potential for transmission i.e. imported cases and suitable vectors, foci IRS and health education are recommended. In type 3 foci, without transmission i.e. imported cases but without suitable vectors or with suitable vectors but not in the transmission season, health education is the recommended response [42]. This step of focus investigation and response is often conducted in a non-standardised manner by local healthcare professionals as it has been reported that while in certain locations, they preferred conducting indoor residual spraying (IRS) even in pseudo-active foci. In other locations, healthcare professionals were hesitant to carry out IRS due to fears of causing panic in the local community [41]. Compliance of the local community created additional difficulties [41]. This is likely to become an even bigger problem as case numbers continue to decline and the public health focus on malaria is diminished. The same lack of standardisation has been observed in determining the radius over which to intervene as part of focus investigation and response. Moreover, practices regarding RACD in foci were also variable, with most relying on RDTs [22], given the absence of molecular techniques such as PCR and LAMP, and the latter having a high false negative rate for asymptomatic cases. Determining the range of foci response activities is also a challenge especially in suburb areas with larger populations. This will likely also be limited by staff experience and knowledge of malaria elimination strategies. A survey circulated among healthcare staff revealed that significantly fewer could correctly identify the classes of malaria cases and types of transmission focus than those who correctly identified different types of case detection methods [38].

In Cambodia, decreased staff motivation to lead foci investigation activities was found to be a hindrance, especially during public holidays [9]. Furthermore, the value of extensive foci investigation, especially in areas of low transmission was questionable, particularly when the previous case investigation step suggested household transmission was minimal.

In Vietnam, reported challenges included inability to contact index cases and unwillingness of asymptomatic contacts to undergo testing for RACD activities [33].

### Adaptations

China implemented the 1-3-7 strategy as part of their national malaria elimination plan in 2012, as case reporting within 1 day, case investigation within 3 days and focus investigation and response within 7 days. Soon after, other countries within the region implemented the strategy, though local logistics led to modifications of the strategy during implementation.

In Thailand, similar to China, healthcare staff are required to report cases within 24 h through the Malaria Information System (MIS). Case investigation in 3 days has been adapted to determine whether the cases are indigenous or imported. Focus investigation is also carried out similar to in China, but classifies foci into unsuitable or suitable for transmission in 4 groups (active foci (indigenous cases within the current year), residual non-active foci (indigenous cases within the past 3 years, but none in the current year), cleared foci but receptive (without indigenous cases in the past 3 years but vectors present/environment suitable for vector breeding), cleared foci but not receptive (without cases in the past 3 years and no vectors found/environment not suitable for vector breeding) [23], as opposed to the 3 in China [31, 41]. Furthermore, in Thailand, only confirmed positive cases would trigger surveillance as opposed to all suspected cases as in China, likely due to the more limited availability of resources. Importantly, the last step of the strategy has been more leniently implemented in Thailand with the possible investigation of foci within 14 days following case reporting counting as within the timeline [31].

In Myanmar [32], the strategy was applied in a very similar way to in China. Patients with fever of unknown cause are tested with RDT/microscopy with treatment provided to positive patients and the township Vector Borne Disease Control team notified within 24 h. Case investigation and classification are then carried out within 3 days and response with complete treatment of index cases, indoor insecticide spraying, active case detection and focus investigation within 7 days, though the latter was yet to be implemented at the time of the studies included in this review.

In Cambodia, the strategy was officially implemented as a 1-3-7 model, with suspected cases identified by village health workers for confirmation by RDT/microscopy within 24 h, investigation of the case (e.g. species, travel status, indigenous/imported) within 3 days, and targeted response measures within 7 days including reactive case detection and insecticide treated nets. That case investigation and response activities were often conducted at the same time, with a 1-2-2 or 1-3-3 model [9], was done in part because it was found that local transmission was rare with case investigations often revealing no household transmission, possibly also indicating that extensive focus investigation was not the most efficient use of resources. In addition to the 1-3-7 strategy, follow up microscopy would be conducted specifically for the *P. falciparum* and mixed infections with *P. falciparum* and another species cases at 28 days.

In India, the strategy was implemented as a 1-3-7-14 model which included diagnosis and treatment within 3 days, focus investigation within 7 days and response activities e.g. vector control and radical treatment with primaquine within 14 days [24].

In Vietnam, the strategy was initially implemented as a 2-3-7 model, with case reporting within 2 days, case investigation within 3 days and focus investigation within 7 days in 2016. In 2021, an update by the Vietnam Ministry of Health recommended case reporting and investigation within 2 days and focus investigation within 7 days [33].

In Tanzania, the strategy was similar to in China, defined as case notification within 24 h, household visit within 3 days and focus investigation within 7 days [34].

## Discussion

The 1-3-7 strategy was implemented nationally in China since 2012, and as of 2021, China has been declared malaria-free. Yet China continues to report imported cases frequently; a situation exacerbated by ongoing transmission in the neighbouring malaria-endemic countries. The 1-3-7 policy, or its derivatives, have been implemented with varying degrees of success in other countries.

Since implementation, cases in India, Cambodia and Thailand have significantly declined. The success of this policy was evidenced particularly in Sampov Loun, Cambodia, where since its introduction, there was good compliance coinciding with a rapid decline in malaria and eventually resulting in its elimination from the district, whilst cases still remain elsewhere in the country. However, in Myanmar, cases have not been substantially impacted, suggesting the 1-3-7 has had limited effects with a range of challenges requiring more flexible solutions depending on existing infrastructure. For example, Myanmar had greater difficultly achieving compliance with reporting due to poor mobile phone connectivity.

The true contribution of the 1-3-7 policy to these reductions in malaria cases is difficult to quantify due to a variety of other concurring interventions and policies, including bed net distribution, strengthened surveillance, roll-out of community health workers and wider use of effective antimalarials, as well as economic development and urbanisation, and as such it was not possible to quantify the true impact of the 1-3-7 strategy from published studies.

For example, cases in China were already declining since the 1980s, with 20 cases per million in 2000 and 6 cases per million by 2010. This decline since the 1980s happened against a background of increased use of antimalarials, insecticide treated bed nets and indoor residual spraying [35]. Following the implementation of the 1-3-7 strategy, case numbers continued falling, leading to zero indigenous cases being reported from 2017 [40]. The 1-3-7 strategy has often been quoted as a significant contributor to this achievement, and indeed the number of indigenous cases has decreased significantly since 2010 [40].

Likewise in India, the transfer to a digitalised reporting system allowed quicker interventions in areas with high malaria incidence, as well as better surveillance and more accurate data analysis, in addition to their 1-3-7-14 strategy [24].

A variety of challenges were identified. These included staff-related, including knowledge or experience gaps and shortages; equipment-related, including availability or transportation; or process-related, such as a lack of standardisation or compliance by region, or attributed to difficulty determining patient history thus limiting contact tracing, and determining the origin of infections. Sustainability of the policy is also an emerging challenge as maintaining vigilance despite low case numbers is demanding, particularly in terms of community engagement and awareness, and professional healthcare training and understanding.

Staff and resource limitations were particularly problematic in the border provinces of China which saw high numbers of imported cases. Indeed, in China and Cambodia, since the implementation of the 1-3-7 policy, while indigenous cases reduced, there was a redistribution of cases towards border regions [9, 25, 28] where socioeconomic demographics may differ with greater poverty, reduced access to healthcare services and health education, leading to reduced awareness and reluctance to seek healthcare [27]^.^ This remains a significant challenge to the 1-3-7 policy and an area for future research to better understand and find solutions.

Implementing the 1-3-7 strategy relies on availability of healthcare equipment, trained healthcare personnel and adequate logistics including infrastructure and transport links. These are not available in many high malaria transmission areas, and the problem may worsen during particular times of the year, for various reasons, including changing travel patterns of mobile workers, monsoon rain season.

For example, in Vietnam, time wasted during transport of staff to remote areas led to significant delays in case investigation [33]. India has had greater success with the implementation of its modified 1-3-7-14 strategy after the introduction of geo-tagged Android tablets [24].

The WHO recommends reactive strategies like the 1-3-7 strategy for malaria elimination in extremely low transmission settings with cases nearing zero, where cases would be expected to cluster in endemic areas [43]. This seems to be the case in China, where cases are imported and clustered near the border regions where the continued use of the 1-3-7 strategy could also work well to prevent re-emergence post-elimination. Likewise, at the time of introduction of the 1-3-7 strategy in Cambodia in 2015, cases were already declining, though the rate of reduction in cases was higher post-2015 following the implementation of the 1-3-7 strategy [9].

Another group of strategies recommended by the WHO are targeted elimination strategies which include targeted drug administration and testing for identifiable groups e.g. mobile workers returning from malaria endemic areas [43]. This may be a better strategy for resource limited countries who are not yet near elimination. It is also a strategy that may prove to be advantageous when applied in bordering regions of countries with risk of re-emergence due to imported cases.

This review had several limitations. Synthesising data from multiple studies for the assessment of the impact of the strategy was difficult even within the same country due to the high variability in data collection, availability of data, and the different outcomes considered in the different studies conducted in different regions. It was also more difficult to assess the impact of the strategy in countries other than China as fewer studies have so far been conducted in these countries, where the start of the implementation is also more recent. On the other hand, the countries which have recently started implementing the strategy in different regions in a stepwise manner, create the opportunity for controlled studies investigating impact in areas where the strategy has been implemented compared to those where it has not. Finally, due to resource limitations, it was not possible to contact authors of the included studies or other stakeholders to obtain additional information.

Indeed, more research is needed on the impact and implementation of the (adapted) 1-3-7 strategy in these countries as more data becomes available. Furthermore, implementing the strategy in these countries has suffered from limited logistical availability and hence adaptation of the strategy to mitigate these challenges and ways to improve logistics in these regions should be considered. Current data indicates that imported malaria cases are on the rise with bordering countries most affected. More research is required into how the greater risk of transmission in these areas can be reduced.

## Conclusion

Although the effectiveness of the 1-3-7 approach was generally very high, as demonstrated by high rates of adherence, the true impact on malaria burden could not be quantified as it was implemented against a background of broader elimination strategies. Thailand, Myanmar, Tanzania, Cambodia, India and Tanzania adapted the strategy initially introduced in China with modifications and have had variable success in both the impact and effectiveness of implementation of the strategy. The 1-3-7 approach is an elimination strategy only suitable for very low transmission settings and its success is contingent upon infrastructure and staffing, requiring consistent compliance throughout the year and across each region which makes it particularly challenging at international borders.

## Supplementary Information

Supplementary material 1.

Supplementary material 2.

Supplementary material 3.

Supplementary material 4.

Supplementary material 5.

## Data Availability

All data generated or analysed during this study are included in this article and its supplementary information files.
