# Supplementary material for: Systematic review of evidence for the impact and effectiveness of the 1-3-7 strategy for malaria elimination
Source: Malar J. 2024 Dec 18;23:371. doi: 10.1186/s12936-024-05200-w (PMC11657205; doi:10.1186/s12936-024-05200-w)
Supplement: Supplementary file 2 — Supplementary material 2. [file 12936_2024_5200_MOESM2_ESM.docx]

**Database Search**

**Search terms:**

*(Malaria OR paludism) AND (eliminat* OR eradicat* OR 1-3-7 OR “reactive case detection” OR RACD)*

*Revised search terms: (Malaria OR paludism OR Plasmodium OR falciparum OR vivax) AND ( 1-3-7 OR “reactive case detection” OR RACD OR TRACK OR TRACE OR TRACING)*

**Search fields:**

Title + Abstract + Keywords + MeSH terms (For databases where this is not possible, the strategy must be documented)

**Databases:**

Cochrane Infectious Diseases Group Specialized Register; Cochrane Central Register of Controlled Trials (CENTRAL); MEDLINE; EMBASE; CABS Abstracts; LILACS; Global Health; Medrxiv, Biorxiv.

**Detailed Search Strategies**

- Cochrane

(Malaria OR paludism OR Plasmodium OR falciparum OR vivax) AND ("1-3-7" OR “reactive case detection” OR RACD OR TRACK OR TRACE OR TRACING) with Cochrane - abstract; title; keyword

- Journals@Ovid Full Text <April 16, 2024>

Embase <1996 to 2024 Week 15>

Ovid MEDLINE(R) ALL <1946 to April 15, 2024>

((Malaria or paludism or Plasmodium or falciparum or vivax) and (1-3-7 or "reactive case detection" or RACD or TRACK or TRACE or TRACING)).ab. or ((Malaria or paludism or Plasmodium or falciparum or vivax) and (1-3-7 or "reactive case detection" or RACD or TRACK or TRACE or TRACING)).ti. or ((Malaria or paludism or Plasmodium or falciparum or vivax) and (1-3-7 or "reactive case detection" or RACD or TRACK or TRACE or TRACING)).ko. or ((Malaria or paludism or Plasmodium or falciparum or vivax) and (1-3-7 or "reactive case detection" or RACD or TRACK or TRACE or TRACING)).kf.

- CABS Abstracts

[[[Publication Title: malaria] OR [Publication Title: paludism] OR [Publication Title: plasmodium] OR [Publication Title: falciparum] OR [Publication Title: vivax] OR [ab: malaria] OR [ab: paludism] OR [ab: plasmodium] OR [ab: falciparum] OR [ab: vivax]] AND [[Publication Title: 1-3-7] OR [Publication Title: "reactive case detection"] OR [Publication Title: racd] OR [Publication Title: track] OR [Publication Title: trace] OR [Publication Title: tracing] OR [ab: 1-3-7] OR [ab: "reactive case detection"] OR [ab: racd] OR [ab: track] OR [ab: trace] OR [ab: tracing]]]

- LILACs

(malaria OR paludism OR plasmodium OR falciparum OR vivax) AND ( 1-3-7 OR “reactive case detection” OR racd OR track OR trace OR tracing) AND ( db:("LILACS") AND la:("en")) AND (year_cluster:[2009 TO 2024]) AND (year_cluster:[2009 TO 2024])

- English - title; abstract;; subject

- Global Health

(TI 1-3-7 OR “reactive case detection” OR RACD OR TRACK OR TRACE OR AB 1-3-7 OR “reactive case detection” OR RACD OR TRACK OR TRACE) AND (S1 AND S2)

- Biorvix / medRvix

<https://www.biorxiv.org/search>

for abstract or title "(Malaria OR paludism OR Plasmodium OR falciparum OR vivax) AND ( 1-3-7 OR “reactive case detection” OR RACD OR TRACK OR TRACE)"

for term "for abstract or title "(Malaria OR paludism OR Plasmodium OR falciparum OR vivax) AND ( 1-3-7 OR “reactive case detection” OR RA" and abstract or title "(Malaria OR paludism OR Plasmodium OR falciparum OR vivax) AND ( 1-3-7 OR “reactive case detection” OR RACD OR TRACK OR TRACE)" (match all words)
